# N-butylidenephthalide Attenuates Alzheimer's Disease-Like Cytopathy in Down Syndrome Induced Pluripotent Stem Cell-Derived Neurons

**DOI:** 10.1038/srep08744

**Published:** 2015-03-04

**Authors:** Chia-Yu Chang, Sheng-Mei Chen, Huai-En Lu, Syu-Ming Lai, Ping-Shan Lai, Po-Wen Shen, Pei-Ying Chen, Ching-I Shen, Horng-Jyh Harn, Shinn-Zong Lin, Shiaw-Min Hwang, Hong-Lin Su

**Affiliations:** 1 Program in Tissue Engineering and Regenerative Medicine, Agricultural Biotechnology Center, National Chung-Hsing University, Taichung, Taiwan; 2 Department of Chemistry, Agricultural Biotechnology Center, National Chung-Hsing University, Taichung, Taiwan; 3 Department of Life Sciences, Agricultural Biotechnology Center, National Chung-Hsing University, Taichung, Taiwan; 4 Center for Neuropsychiatry, China Medical University and Hospital, Taichung, Taiwan; 5 Department of Neurosurgery, China Medical University Beigang Hospital, Yunlin, Taiwan; 6 Department of Neurosurgery, Tainan Municipal An-Nan Hospital-China Medical University, Tainan, Taiwan; 7 Graduate Institute of Immunology, China Medical University, Taichung, Taiwan; 8 Department of Pathology, China Medical University Hospital, Taichung, Taiwan; 9 Bioresource Collection and Research Center, Food Industry Research and Development Institute, Hsinchu, Taiwan

## Abstract

Down syndrome (DS) patients with early-onset dementia share similar neurodegenerative features with Alzheimer's disease (AD). To recapitulate the AD cell model, DS induced pluripotent stem cells (DS-iPSCs), reprogrammed from mesenchymal stem cells in amniotic fluid, were directed toward a neuronal lineage. Neuroepithelial precursor cells with high purity and forebrain characteristics were robustly generated on day 10 (D10) of differentiation. Accumulated amyloid deposits, Tau protein hyperphosphorylation and Tau intracellular redistribution emerged rapidly in DS neurons within 45 days but not in normal embryonic stem cell-derived neurons. N-butylidenephthalide (Bdph), a major phthalide ingredient of *Angelica sinensis*, was emulsified by pluronic F127 to reduce its cellular toxicity and promote canonical Wnt signaling. Interestingly, we found that F127-Bdph showed significant therapeutic effects in reducing secreted Aβ40 deposits, the total Tau level and the hyperphosphorylated status of Tau in DS neurons. Taken together, DS-iPSC derived neural cells can serve as an ideal cellular model of DS and AD and have potential for high-throughput screening of candidate drugs. We also suggest that Bdph may benefit DS or AD treatment by scavenging Aβ aggregates and neurofibrillary tangles.

Alzheimer's disease (AD) is the most common neurodegenerative disorder in the elderly. The clinical features of AD patients include progressive memory loss and cognitive impairment, and the histopathological hallmarks in the brain are extracellular amyloid plaques and intracellular neurofibrillary tangles^1^. Extracellular amyloid plaques are composed of aggregated amyloid-β (Aβ) peptides, which are produced from the abnormal splicing of amyloid precursor protein (APP) by β and γ secretases^2^. Genetic mutations in APP or β/γ secretases contribute to the risk of an early onset of AD^3^,^4^,^5^. Neurofibrillary tangles are mainly generated by hyperphosphorylated Tau, a microtubule-associated protein, for intensive microtubule aggregation^6^. The overexpression of Tau and redistribution of the protein from axons to soma or dendrites represent additional unique characteristics of AD cytopathy and may disturb the neurite architecture of affected neurons^7^.

In addition to familial AD, early-onset AD-like dementia is also observed in patients of Down syndrome (DS)^8^, which is the most common hereditary disease and causes mental retardation in one of eight hundred births^9^. Because these individuals carry an extra copy of chromosome 21, harboring the APP^10^ and Dyrk1A kinase (Tau protein phosphorylation kinase) genes^11^, DS patients exhibit an AD-like histopathology in their brain, including amyloid plaque accumulation and neurofibrillary tangles^12^.

Human induced pluripotent stem cells (iPSCs) share similar features of embryonic stem cells and have become a potential *in vitro* cell model of human diseases. Somatic mature cells, such as fibroblast cells, can be reprogrammed into iPSCs by the forced expression of pluripotent genes^13^. Patient-derived iPSCs can further differentiate into specific cell lineages to recapitulate the developmental processes of disease and pathogenesis.

To provide a novel platform for drug screening and disease mechanism exploration for AD^14^,^15^,^16^,^17^,^18^,^19^,^20^,^21^,^22^, the iPSC model of AD has been reported^17^,^18^,^19^. However, in some cases, especially for non-familial AD patients, the iPSCs do not show the classic cytopathological features of AD^17^. In contrast, recent evidence demonstrates that iPSCs from DS patients (DS-iPSCs) faithfully recapitulate the molecular signatures of AD *in vitro*^20^,^22^,^23^. In this study, we used the BiSF method, an efficient and rapid neural induction procedure^24^, to differentiate DS-iPSCs into neuroepithelial progenitor cells (NPCs) and mature forebrain neurons. The paradigmatic cytopathology of AD was observed in our DS-iPSC-derived neurons within a short period of time. We also applied this *in vitro* model for drug screening and found that a small molecule extracted from natural herbs shows a therapeutic effect in reducing Aβ accumulation, Tau protein expression and Tau hyperphosphorylation.

## Methods

### hESC and hiPSC cultures

hESC and iPSC lines, including the TW1 hES line (from Lee Women's Hospital, Taiwan)^25^ and allantoic fluid-derived trisomy 21 iPS cells (from Food Industry Research and Development Institute, Taiwan)^26^, were cultured in Essential 8 medium (Invitrogen, Carlsbad, CA, USA) on 1.0% hES qualified Matrigel (Becton Dickinson, BD, Franklin Lakes, NJ, USA)-coated cell culture dishes. The establishment of both TW1 hESCs and DS-iPSCs followed the Policy Instructions on the Ethics of Human Embryo and Embryonic Stem Cell Research in Taiwan. In addition, approval from the Ethic Institutional Review Board and informed consent were also obtained, as described in previous reports^25^,^26^. The cells were passaged for 3–5 days using Accutase (Merck-Millipore, Billerica, MA, USA) and mechanical scraping and were then reseeded at a 1:5 or 1:10 ratio. The culture medium was refreshed daily.

### Neural induction and neuronal maturation

When cell confluence was more than 80% in a culture dish, the cells were treated with Accutase for 2–5 minutes and harvested by scraping. The cell clumps were dissociated into 200–300 μm clusters and transferred to non-coated Petri dishes for 2 days. The isolated cells were subjected to BiSF neural induction. Briefly, the differentiation medium within the first two days was DMEM-F12 basal medium (Invitrogen) supplied with 20% knockout serum replacement (KSR, Invitrogen), 1 mM non-essential amino acids (NEAAs, Invitrogen), 2 mM glutamate (Invitrogen) and 0.1 mM 2-mercaptoethanol (Invitrogen). Spherical cell aggregates were formed at this stage. On D3 of differentiation, the cell culture medium was changed to DMEM-F12 supplied with 1% N2 supplement (Invitrogen), 1 mM NEAAs and 2 mM glutamate. Neural-inducing factors, including 0.5 μM BIO (Sigma-Aldrich), 10 μM SB431542 (Sigma-Aldrich, St. Louis, MO, USA) and 10 ng/mL recombinant human FGF-2 (rh-FGF2, R&D Systems, Minneapolis, MN, USA) were added on D3 for 2 days^24^. On D5, the neural induction medium was removed, and the neurospheres were cultured in neurobasal medium (Invitrogen) with 1% N2 supplement and 10 ng/mL rh-FGF2.

After neural induction, the cells were dissociated into small clumps by Accutase and seeded on 1% Matrigel (Invitrogen)-coated cell culture dishes for neural maturation. Initially, the NPCs proliferated in neurobasal medium with 2% B27 supplement (Invitrogen) and 10 ng/mL rh-FGF2. During the passaging of NPCs with Accutase, providing 10 μM Y27632 (Sigma-Aldrich) effectively attenuated dissociation-triggered cell death. For driving neural maturation, the cells were maintained for weeks in neurobasal medium without rh-FGF2 from D15.

### Immunocytochemistry (ICC) staining

Cells seeded in 4-well plates were fixed with 4% paraformaldehyde (Sigma-Aldrich) and washed twice with phosphate-buffered saline (PBS). The cells were permeabilized with 0.3% Triton-100 for 10 min and then treated with 5% horse serum. Primary antibodies were added and incubated at 4°C overnight. The primary antibodies used in this study included those against Sox-1 (1:200, Santa Cruz Biotechnology, Dallas, TX, USA), Pax-6 (1:100, Covance, Princeton, NJ, USA), Nestin (1:500, Covance), N-cadherin (1:500, Santa Cruz Biotechnology), Forse-1 (1:50, Development Studies Hybridoma Bank, DSHB, IA, USA), βIII-tubulin (TuJ1, 1:500, Covance), MAP2 (1:1000, Merck Millipore), phospho-PHF-Tau pSer^202^/Thr^205^ (AT8, 1:200, Thermo Scientific, Rockford, IL, USA), β-Amyloid 1-42 (Aβ42, 1:500, Merck Millipore) and β-catenin (1:500, BD). The cell nuclei were stained with diamidino-2-phenylindole (DAPI). Fluorescence images were captured using an upright microscope (Eclipse TE2000-S and 80i, Nikon, Tokyo, Japan) or a confocal microscope (LSM 510, Carl Zeiss, Oberkochen, Germany).

### Extracellular protein measurement by sandwich ELISA

After neuronal differentiation, conditioned medium was collected from 4 × 10^5^ cells and centrifuged at 1000 rpm for 5 min. The supernatants were stored at −80°C immediately as extracellular samples. These collected samples were subjected to sandwich ELISA analysis for quantitatively measuring Aβ40 (Covance), Aβ42 (Covance), human total Tau (Invitrogen) and human Tau pS^396^ (Invitrogen).

### Synthesis of aqueous n-butylidenephthalide (Bdph)

Bdph (Sigma-Aldrich) and Pluronic F127 (Sigma-Aldrich) were mixed at a ratio of 1:1, and the mixtures were dissolved in tetrahydrofuran. The complex was dissolved in water and heated at 85°C to evaporate the tetrahydrofuran. The F127-encapsulated Bdph was concentrated by freezing and drying^27^.

### Analysis of F127-Bdph

The size distribution of the F127-Bdph formulation was analyzed by dynamic light scattering (ZS90, Malvern Instruments, Worcestershire, UK) at 25°C. The samples for TEM analysis were prepared by spreading a drop of the diluted F127-Bdph solution on carbon-coated 200 mesh copper grids. The samples were dried in air and then observed under a transmission electronic microscope (TEM, JEM 1400, JEOL, Tokyo, Japan) with an accelerating voltage of 120 kV.

### MTT proliferation assay

Confluent cells were grown in 96-well plates and incubated with 100 μL 1 mg/ml 3-(4,5-cimethylthiazol-2-yl)-2,5-diphenyl tetrazolium bromide (MTT, USB, Cleveland, OH, USA) at 37°C for 4 hrs. The MTT dissolved in DMSO (100 μL) was measured using an ELISA reader (Beckman-Coulter, Brea, CA, USA) at 540 nm.

### TCF luciferase reporter assay

Human neuroblastoma NT2 cells were cultured in Opti-MEM (Invitrogen) with 10% fetal bovine serum (FBS, Invitrogen), 1 mM NEAA (Invitrogen), and 2 mM glutamate (Invitrogen). TCF reporter plasmids, including TOP-Flash and control FOP-Flash (both from Merck-Millipore), were transfected into the cells using Lipofectamine 2000 (Invitrogen). The transfection efficacy was estimated by cotransfecting a plasmid carrying green fluorescent protein (GFP). At 24 h post-transfection, the cells were treated with F127-Bdph or other factors for 3 days. The luciferase activities were measured by a firefly luciferase of luciferase reporter assay (Promega, Madison, WI, USA), and the luciferase activities were analyzed with an ELISA reader (Beckman-Coulter).

### Statistical analysis

Data were collected from more than three independent experimental results and are shown as the mean value ± SD. Statistical analyses were conducted using Student's *t* test between two groups or a one-way ANOVA with a Tukey's *post hoc* test. A *p* value less than 0.05 was considered to be significant.

## Results

### Robust neural differentiation of DS-iPSCs by the BiSF method

**Figure 1:**
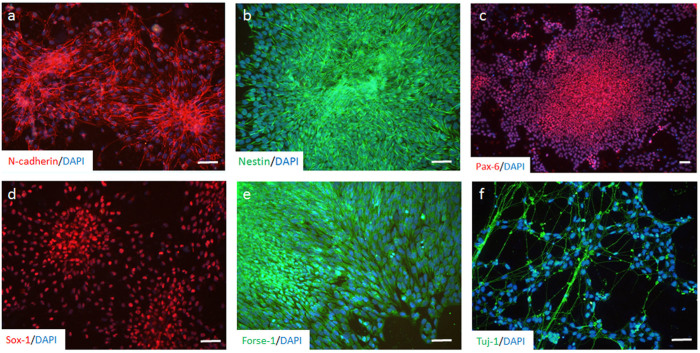
BiSF method drives efficient neural differentiation of DS-iPSCs. (a–d) BiSF-treated DS-iPSCs expressed NPC-specific markers, including N-cadherin (a), Nestin (b) and Pax-6 (c) on D10 and Sox-1 (d) on D15. In addition, forebrain marker Forse-1 (e) on D10 and mature neuronal marker β-III tubulin (stained by Tuj1 antibody, f) on D25 were also detected. Scale bar, 50 μm.

We first applied the BiSF neural induction method to investigate the efficacy of the neural differentiation of DS-iPSCs. The results of ICC staining showed that more than 90% of the DS-iPSCs cells were converted to a neural cell fate on D10 of differentiation, with the expression of NPC markers, such as N-cadherin and Nestin (Fig. 1a and 1b). Two NPC-specific transcription factors, Pax-6 and Sox-1, were also robustly expressed on D10 and D15, respectively (Fig. 1c and 1d). We also detected a forebrain-specific antigen, Forse-1, in most of the NPCs (Fig. 1e). Removing FGF2 from the culture medium from D15 decreased cell proliferation but promoted cell differentiation and neurite formation. Extensive neurite extension and the arborization of mature neurons were detected with the expression of a neurite-specific protein, β-III tubulin (stained by the Tuj-1 antibody), on D25 (Fig. 1f). These results demonstrate that DS neurons with high purity and forebrain characteristics can be efficiently and rapidly converted from pluripotent DS-iPSCs.

### DS-iPSC-derived neurons generate accumulated amyloid plaques

**Figure 2:**
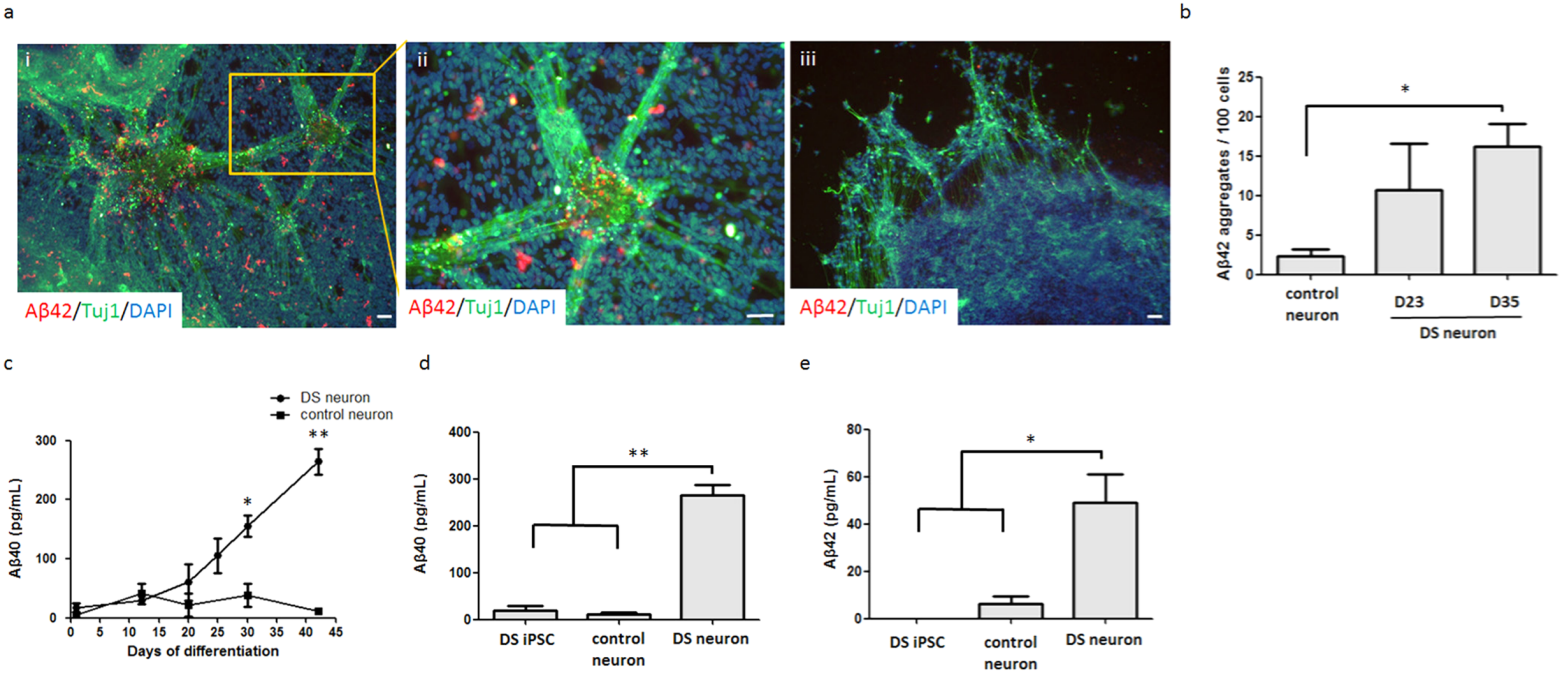
DS neurons produce amyloid aggregates. (a) Aβ42 aggregates were measured in DS neurons (i and ii) and TW1-derived normal neurons (control, iii) by ICC staining with an anti-Aβ42 antibody (red) and neuronal marker β-III tubulin (green) on D35. Cell nuclei were labeled with DAPI (blue). (b) Aβ42 aggregate numbers on D23 and D35 were calculated and normalized to the total cell number. (c) Culture media were collected and analyzed with an Aβ40 ELISA kit at the indicated times. (d, e) The ELISA data for Aβ40 (d) and Aβ42 (e) on D42 are illustrated, including the results of undifferentiated DS-iPSCs, TW1-derived neurons (control) and DS neurons. The data represent triplicate experimental results. Statistical results were analyzed with a one-way ANOVA, and the significance was examined by Tukey's post-hoc assay. *, *p* < 0.05. **, *p* < 0.01. Scale bar, 50 μm.

Amyloid plaques, a hallmark of AD cytopathy, are primarily formed by Aβ40 and Aβ42^28^. The differentiating DS-iPSCs first exhibited insoluble Aβ42 aggregates on D20 of neural differentiation. Extracellular amyloid aggregates were intensively formed from the differentiating DS-iPSCs on D35 (16.23 ± 5.05 aggregates per 100 cells), whereas normal hESC-derived neurons were almost free of these aggregates at the same stage (2.28 ± 1.51 aggregates per 100 cells) (Fig. 2a and 2b).

Recent experiments illustrate that only the neurons derived from DS or AD patients exhibit the AD like cytopathology, but not the neurons from healthy donor-derived hESCs and iPSCs^16^,^17^,^18^,^20^,^23^,^26^. Accumulated evidence also indicates that both hESCs and iPSCs, derived from healthy people, share high similarity in self-renewal and potency of differentiation^24^,^29^,^30^. These findings support the use of hESCs and their derivative neurons as a control for our DS-iPSC experiments.

In addition to insoluble amyloid plaques, soluble Aβ in the supernatant of the culture medium was detected by ELISA, and Aβ40 secreted from DS neurons was significantly increased on D30 compared to the normal control neurons (DS neurons, 155.68 ± 18.51 pg/mL; control neurons, 39.09 ± 19.61 pg/mL) (Fig. 2c). The DS neurons secreted much more Aβ40 (264.47 ± 22.16 pg/mL) into the medium on D42, in contrast to the healthy control neurons (10.91 ± 4.18 pg/mL) and undifferentiated DS-iPSCs (19.77 ± 9.00 pg/mL) (Fig. 2d). The control neurons only secreted a limited amount of Aβ40 into the medium during neuron maturation. We also observed that the expression kinetics of Aβ42 secreted from the DS neurons shared a pattern similar to that of Aβ40. Aβ42 was strongly released from DS neurons on D42 (48.93 ± 24.43 pg/mL) compared to control neurons (6.07 ± 7.05 pg/mL) or undifferentiating DS-iPSCs (almost undetectable) (Fig. 2e). These data illustrate that DS-iPSCs differentiating according to our BiSF neural differentiation protocol rapidly and faithfully recapitulate amyloid formation *in vitro*.

### Hyperphosphorylation and redistribution of Tau protein in DS neurons

**Figure 3:**
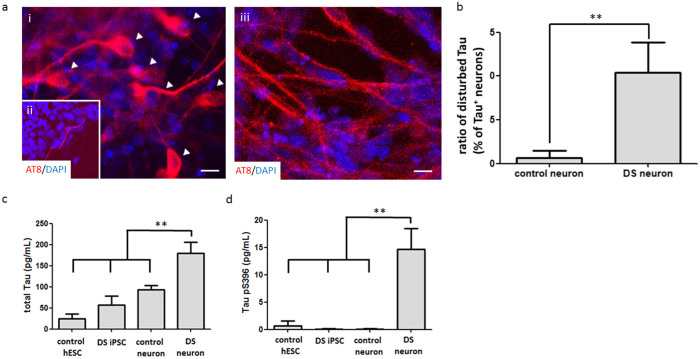
The redistribution and hyperphosphorylation of Tau protein were detected in DS neurons. (a) DS neurons (i and ii) and control neurons (iii) were analyzed with an anti-AT8 antibody (red), which recognizes phosphorylated Tau. Activated Tau was distributed in the cell soma (i, arrowhead) and entire cell (ii) of DS neurons rather than in the axons of the control neurons (a-iii) on D42. The ratio of disturbed Tau in total mature neurons was calculated according to the ICC staining (b). (c, d) Released total Tau (c) and pS^396^ Tau (d) in media were subjected to an ELISA analysis on D43 and D45, respectively. Control hESCs (TW1) and DS-iPSCs were in an undifferentiated status. **, *p* < 0.01. Scale bar, 10 μm.

The hyperphosphorylation and redistribution of Tau protein from the axon to cell body or dendritic trees are also cytopathic features of AD. Tracing Tau phosphorylated at Ser^202^ and Thr^205^ in DS neurons illustrated the relocalization of activated Tau from the axon to the cell soma (Fig. 3a-i). In particular, activated Tau was distributed throughout the entire cell in some DS neurons (Fig. 3a-ii) and not restricted to the axons, as normal neurons (Fig. 3a-iii). Statistical analysis of the immunocytostaining further revealed that the Tau redistribution ratios in control neurons and DS neurons were 0.68 ± 0.78% (n = 657) and 10.40 ± 3.49% (n = 736), respectively (*p* < 0.01) (Fig. 3b).

Clinical studies have indicated that increased levels and hyperphosphorylated Tau protein in the cerebrospinal fluid (CSF) of AD patients could be used as AD biomarkers for early diagnosis^31^. In our model, we also detected that the level of Tau protein was dramatically upregulated in the medium of DS neurons (179.36 ± 26.73 pg/mL) compared to that from control TW1 hESC-derived neurons (93.23 ± 9.55 pg/mL), undifferentiated DS iPSCs (56.86 ± 21.49 pg/mL) and TW1 hESCs (24.82 ± 11.62 pg/mL) (Fig. 3c). We also noted that a unique phosphorylated Tau at Serine 396 (Tau pS^396^) in AD-affected neurons, targeted by active GSK-3β, was consistently detected in the culture medium of DS neurons on D45 (14.71 ± 3.71 pg/mL) but not in that of the control neurons at the same differentiation stage (Fig. 3d).

### Pluronic-F127-coated n-butylidenephthalide (Bdph) activates the Wnt signaling pathway

**Figure 4:**
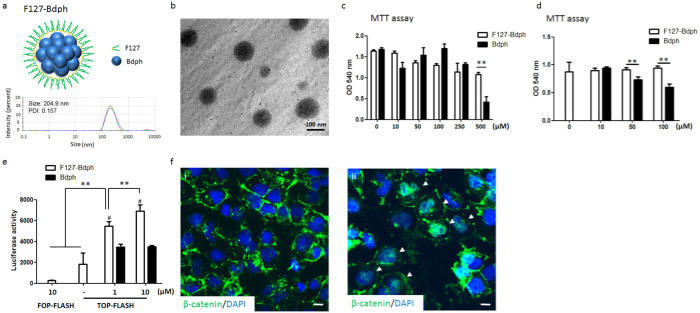
F127-Bdph activates Wnt signaling pathway. (a) Structure illustration and size distribution of the F127-Bdph composite. (b) TEM image of F127-Bdph. (c, d) NT2 cells (c) and DS neurons (d) were treated with Bdph and F127-Bdph for 3 days and 5 days respectively, and cell viability was measured by the MTT assay. (e) NT2 cells, pre-transfected with FOP-Flash or TOP-Flash plasmids, were treated with Bdph or F127-Bdph for 3 days. The luciferase level represents the Wnt/TCF transcriptional activity. (f) PBS alone- (i) and F127-Bdph-treated (10 μM for 3 days, ii) NT2 cells were analyzed with β-catenin ICC staining. The translocated β-catenin in the nucleus is indicated by arrow heads. *, *p* < 0.05. **, *p* < 0.01. #, *p* < 0.01, pair comparison between Bdph and F127-Bdph treated cells at 1 and 10 μM. Scale bar, 10 μm.

Certain phthalide analogs have shown neuronal protection activity in brain injury and neurodegenerative diseases^32^,^33^,^34^,^35^,^36^,^37^. As a major component of the Chinese herb *Angelica sinensis*, n-butylidenephthalide (Bdph) was tested for its ability to reduce amyloid aggregates in our DS-iPSC model. We first observed a high cytotoxic effect of Bdph (10 μM–50 μM) on hESC-derived neurons by morphological observation. To reduce this toxicity, pluronic-F127, a thermo-sensitive hydrogel^38^, was used to encapsulate Bdph for slowed release^27^. As shown in Fig. 4a, F127-Bdph complex exhibited a size around 200 nm with a narrow polydispersity index, measured by dynamic light scattering. Spherical shape of F127-Bdph nanoparticles was observed by TEM (Fig. 4b). Cytotoxic analysis of the Bdph treated cells, determined by MTT assay, illustrated that the coating with F127 significantly attenuated the cytotoxicity of Bdph in both human neuroblastoma NT2 cells (Fig. 4c) and DS-iPSC derived neurons (Fig. 4d).

Accumulated evidence indicates that dysfunction in Wnt signaling is associated with AD progression, while the restoration of Wnt activation ameliorates AD cytopathy. To measure the Wnt activation capacity of F127-coated Bdph, both the transcriptional activation of TCF and nuclear translocation of β-catenin were determined. NT2 cells were transfected with a Wnt signal reporter, a TCF luciferase reporter plasmid (TOP-Flash), or a control reporter, mutant FOP-Flash, before treatment with F127-Bdph. We found that F127-Bdph dose-dependently activated TCF-driven luciferase activities after three days of incubation (1 μM, 5458.00 ± 446.41; 10 μM, 6891.00 ± 621.62) but not in the untreated cells (1839.33 ± 1057.43) and cells carrying FOP-Flash (283.33 ± 40.65) (Fig. 4e). Comparing to Bdph alone, the F127 coating potentiated the TCF-driven luciferase activities of Bdph in NT2 cells (Fig. 4e). These results indicate that F127-mediated emulsification not only enhances the cell compatibility of Bdph but also preserves the Wnt bioactivity of Bdph. Furthermore, nuclear β-catenin translocation was frequently observed in the F127-Bdph-treated NT2 cells (37.79 ± 4.74%, n = 419) but not in non-treated cells (5.38 ± 0.41%, n = 479) (*p* < 0.01, student's *t* test), supporting a role of Bdph on Wnt activation in human neural cells (Fig. 4f).

### F127-Bdph reduces Aβ deposition and Tau protein overexpression and hyperphosphorylation in DS neurons

**Figure 5:**
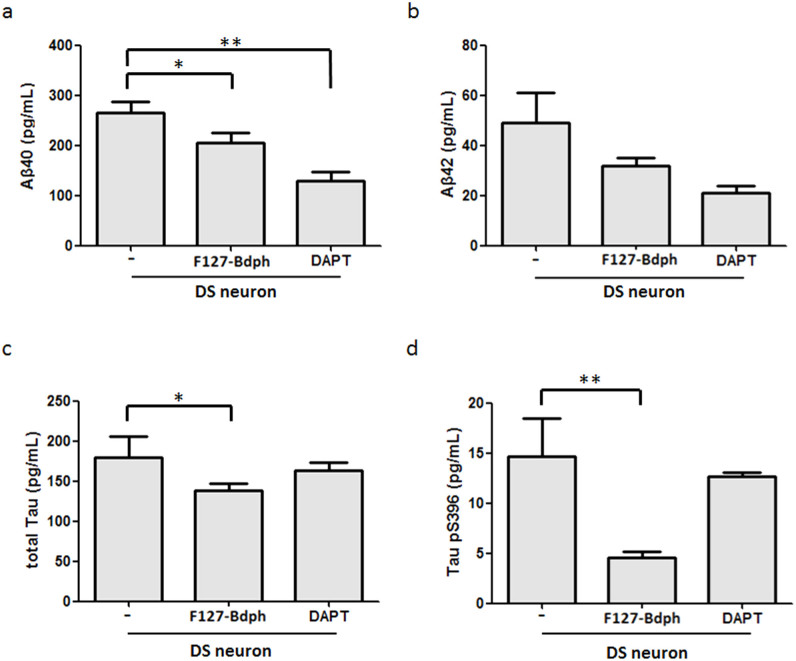
F127-Bdph reduces Aβ production and Tau cytopathy in DS neurons. After treatment with 10 μM F127-Bdph for three days, culture media were collected and subjected to analyses for Aβ40 (a), Aβ42 (b), total Tau (c) and Tau (pS^396^) (d) by ELISA. *, *p* < 0.05. **, *p* < 0.01.

To test whether F127-Bdph has a therapeutic effect on DS neurons, we added 10 μM F127-Bdph to DS neurons and measured the amount of Aβ40, Aβ42, total Tau protein and hyperphosphorylated Tau protein (tau-pS^396^) in the medium. A well-documented γ-secretase inhibitor, *N*-[*N*-(3,5-difluorophenacetyl-L-alanyl)]-(*S*)-phenylglycine *t*-butyl ester (DAPT)^39^, was introduced as a positive control. DAPT treatment attenuated secreted amyloid Aβ40 (128.86 ± 18.78 pg/mL) and Aβ42 (20.87 ± 5.09 pg/mL) by one-half, compared to the untreated group (Aβ40, 264.47 ± 22.16 pg/mL; Aβ42, 48.93 ± 24.43 pg/mL) (Fig. 5a and 5b). However, we discovered that DAPT treatment did not significantly ameliorate the Tau expression and phosphorylation abnormalities (Fig. 5c and 5d). In contrast, F127-Bdph treatment both significantly decreased the production of amyloid peptides (Aβ40, 204.61 ± 20.28 pg/mL; Aβ42, 32.01 ± 6.15 pg/mL; *p* < 0.05) (Fig. 5a and 5b) and reduced Tau cytopathy, including the level of protein expression (179.36 ± 26.73 pg/mL to 137.78 ± 9.73 pg/mL, *p* < 0.05) and hyperphosphorylated Tau in the medium (14.71 ± 3.71 pg/mL to 4.54 ± 0.66 pg/mL, *p* < 0.01) (Fig. 5c and 5d). These results demonstrate that F127-Bdph shares an activity similar to DAPT in reducing amyloid aggregates and also has a stronger therapeutic effect on ameliorating the abnormality of Tau production and hyperphosphorylation in DS neurons.

## Discussion

Here, we demonstrate that the combination of DS-iPSCs and a BiSF neural differentiation method creates an ideal *in vitro* cell model for AD-like cytopathy. Robust forebrain neurons with high purity were rapidly induced on D10. The maturation of DS neurons was accompanied with the formation of amyloid aggregates and Tau abnormality, two major features of AD cytopathy, within 45 days of culture. Moreover, treating the DS neurons with the γ-secretase inhibitor DAPT reproducibly reduced extracellular Aβ40/Aβ42 formation. Compared to previous time-consuming experiments using iPSCs as an *in vitro* AD model^17^,^18^,^20^, our culture method provides a stable and faster neural differentiation system for recapitulating AD-like cytopathology.

In this study, early amyloid aggregates and later Tau overexpression and hyperphosphorylation were consistently detected in DS neurons. The appearance of amyloid deposits and Tau cytopathy is suspected to trigger cell death in affected DS neurons. Interestingly, we noted that reactive oxygen species (ROS) and cell death were not remarkably increased in DS neurons, even in the cells cultured until D60 (data not shown). Although we may observe severe cytopathic effects on DS neurons at a later stage, several possibilities can be proposed for the low cytotoxicity of DS neurons within 60 days culture. We speculate that the picogram level of spontaneously produced Aβ by DS neurons, in contrast to the microgram level of exogenous Aβ for enforcing cell death, is too small to cause dramatic cell stress and neuron degeneration^2^. In addition, the enriched culture medium used for the DS neurons may antagonize the lethal factors of Aβ accumulation and Tau abnormality. A recent study also indicated that the replacement of an enriched culture medium with a starvation medium is necessary to drive ROS production and cell death in AD-iPSC-derived neurons^17^. Moreover, other neural cells, which were absent in our culture system, such as glial cells and microglia, may be critical for initiating neurodegeneration. Interestingly, a recent work demonstrates that DS-iPSC-derived astroglia are over-reactivated, playing a critical role in neuronal cytotoxicity^22^. The interaction between DS neurons and astroglia will be further evaluated to elucidate the pathogenesis of neuronal degeneration.

Persistent Wnt activation is physiologically essential for the maintenance of synapse integrity and neuronal circuits^40^. Conversely, a reduction in Wnt signaling is highly associated with AD progression^40^. Cellular studies of AD indicate that the emergence of extracellular Aβ oligomers disrupts the transduction of Wnt signaling at the neuronal membrane and impairs neural connections, neuronal survival and neurogenesis^41^,^42^,^43^,^44^. Experimental data indicate that restoring Wnt activation by adding Wnt ligands^45^ or inhibitors of Dickkoph-1^44^ and glycogen synthase kinase-3β (GSK-3β)^46^ successfully attenuates Aβ toxicity and repairs cognition performance in animal model of AD.

In particular, GSK-3β, the major intracellular modulator of Wnt signaling, is directly involved in the formation of neurofibrillary tangles^47^. Given that Wnt signaling is downregulated by Aβ deposits, hyperactivated GSK3β not only accelerates Tau protein phosphorylation^47^ but also stimulates activated presenilin-1 and amyloidogenic processing^48^.

In this study, we demonstrated that the Bdph small molecule can activate canonical Wnt signaling, as evidenced by enhanced TCF activity and nuclear β-catenin translocation. The Bdph-treated DS neurons showed a remarkable decrease in total Tau production and hyperphosphorylated Tau protein (pS^396^), which is directly phosphorylated by activated GSK-3β. It is possible that Bdph directly or indirectly inhibits GSK-3β activation to prevent Tau phosphorylation. Elucidating the detail molecular mechanism of Bdph requires further experiments to examine the binding affinity between Bdph and GSK-3β, molecular docking modeling and the phosphorylation status of GSK-3β in Bdph-treated neurons.

In addition to the novel role of Wnt activation revealed in this study, Bdph can inhibit telomerase and modulate Erk and Akt signaling, which are associated with its anti-cancer activity at high doses^49^,^50^,^51^,^52^. Bdph has also been reported to improve dopaminergic neuron degeneration and α-synuclein accumulation in *Caenorhabditis elegans* models of Parkinson's disease^32^, though the underlying mechanism is still unclear. Notably, n-butylphthalide (NBP), which shares the same chemical structure as Bdph, shows strong neuron protection activity in acute brain ischemia and neural degenerative diseases^33^,^34^,^35^,^36^,^37^. Treating AD with NBP activates MAPK^53^ and Akt signaling^34^ and attenuates Aβ accumulation, Tau overexpression and cognitive impairment in AD models. Whether Bdph at a low dose also activates MAPK and AKT signals and whether Wnt activation is also involved in NBP-mediated neural protection are interesting topics for further investigation.

In conclusion, our study demonstrates that DS-iPSC derived neurons represent an ideal *in vitro* model that faithfully recapitulates the classical cytopathies of AD. By monitoring Aβ and Tau protein in the culture medium, we demonstrated that Bdph may be a potential small molecule for controlling the progression of AD or DS dementia. In addition to drug screening, this cell model is also suitable for identifying early pathological biomarkers of AD and delineating molecular profiles during DS progression.

## Author Contributions

S.M.H. and H.L.S. initiated this project. C.Y.C., S.M.C., H.E.L., S.M.H. and H.L.S. conceived and designed the experiments. C.Y.C., S.M.C., H.E.L., S.M.L., P.S.L., P.W.S., P.Y.C. and C.I.S. performed the experiments. P.S.L., H.J.H., S.Z.L. and S.M.H. contributed reagents/materials/analysis tools. C.Y.C., S.M.H. and H.L.S. analyzed the data and wrote the paper. All authors reviewed the manuscript.
